# Duodenal ampullary neuroendocrine tumor, high risk gastrointestinal stromal tumor, and gastric leiomyoma in a patient with neurofibromatosis type 1: a rare case report and literature review

**DOI:** 10.3389/fonc.2026.1738556

**Published:** 2026-02-11

**Authors:** Xiaonan Yin, Hongxin Yang, Yuan Yin, Bo Zhang

**Affiliations:** 1 Gastric Cancer Center, Department of General Surgery, West China Hospital, Sichuan University, Chengdu, Sichuan, China; 2 Department of Gastrointestinal Surgery, The Affiliated Hospital of Guizhou Medical University, Guiyang, Guizhou, China; 3 Department of Gastrointestinal Surgery, West China Hospital, Sichuan University, Chengdu, Sichuan, China

**Keywords:** case report, gastrointestinal stromal tumor, leiomyoma, neuroendocrine tumor, neurofibromatosis type 1

## Abstract

Neurofibromatosis type 1 (NF1), an autosomal dominant disorder resulting from mutations in the *NF1* tumor suppressor gene, predisposes affected individuals to diverse benign and malignant neoplasms. We herein report a rare case of a 61-year-old female NF1 patient presenting with a unique combination of four synchronous or metachronous tumors: a duodenal ampullary neuroendocrine tumor (NET, Grade 2), a high-risk gastrointestinal stromal tumor (GIST) of the small intestine, a previously resected gastric leiomyoma, and cutaneous neurofibromas. Preoperative evaluation included endoscopy, endoscopic ultrasound, contrast-enhanced CT, and MRI, which localized the lesions and assessed their morphological features. The patient underwent pancreaticoduodenectomy and partial small bowel resection, achieving complete tumor resection. Genetic testing identified a germline *NF1* mutation (p.Y2182*). No adjuvant therapy was administered due to the absence of residual disease and actionable mutations. This case highlights the broad tumor spectrum in NF1 and underscores the importance of comprehensive imaging, multidisciplinary management, and genetic testing for optimal outcomes.

## Background

Neurofibromatosis type 1 (NF1), also known as von Recklinghausen disease, is an autosomal dominant disorder caused by mutations in the *NF1* tumor suppressor gene located on chromosome 17q11.2 (1). With an estimated incidence of 1 in 3,000 live births, NF1 ranks among the most prevalent neurocutaneous syndromes (2). Its clinical manifestations include café-au-lait macules, cutaneous neurofibromas, Lisch nodules, and optic pathway gliomas (3). More importantly, NF1 confers an elevated lifetime risk of developing both benign and malignant tumors, particularly those of neural crest origin, such as malignant peripheral nerve sheath tumors (MPNSTs), pheochromocytomas, and gastrointestinal stromal tumors (GISTs) (4).

Although GISTs represent the most common mesenchymal tumors of the gastrointestinal tract and are frequently driven by *KIT* or *PDGFRA* mutations (5), NF1-associated GISTs constitute a distinct subtype. These tumors often arise in the small intestine, are typically multifocal, and usually lack the typical driver mutations seen in sporadic cases (6, 7). They commonly follow an indolent yet progressive clinical course. Neuroendocrine tumors (NETs), though less frequently associated with NF1, have been documented primarily in the duodenum and pancreas (8, 9). Duodenal NETs may present with symptoms related to biliary or pancreatic duct obstruction, such as jaundice or pancreatitis, depending on their location and size. Gastric leiomyomas, benign smooth muscle tumors, are occasionally discovered incidentally during surgical procedures or imaging examinations. The concurrent presence of NETs, GISTs, and leiomyomas in NF1 patients is exceedingly rare. To our knowledge, this represents the first reported case of a synchronous duodenal ampullary NET, a high-risk small intestinal GIST, and a previous gastric leiomyoma occurring in a single NF1 patient.

This case report aims to highlight the rare but important tumor spectrum associated with NF1, emphasizing the need for a high index of suspicion, comprehensive diagnostic workup, and a multidisciplinary approach in the management of such patients.

## Case report

A 61-year-old female was referred to West China Hospital, Sichuan University, in September 2024, presenting with a six-year history of nonspecific upper abdominal discomfort. Her medical history was unremarkable without family history of NF1, or other genetic disorders. Six years earlier, she had undergone proximal gastrectomy and excision of multiple cutaneous nodules for a gastric tumor and coexistent dermatological lesions. Histopathological examination of gastric mass confirmed a diagnosis of leiomyoma, supported by immunohistochemical positivity for smooth muscle actin (SMA) and caldesmon, alongside negativity for CD117, DOG1, and CD34. Examination of the cutaneous lesions confirmed cutaneous neurofibromas, characterized by immunohistochemical expression of S-100 and NF, with absence of SMA and desmin. She was admitted this time due to recurrent upper abdominal discomfort accompanied by nausea and vomiting. Notably, she denied diarrhea, chest tightness, chest pain, or dyspnea. Physical examination revealed no scleral or cutaneous jaundice. The abdomen was soft, without tenderness, rebound tenderness, or guarding. No palpable masses were detected, and Murphy’s sign was negative. Laboratory studies demonstrated elevated alkaline phosphatase (ALP, 168 U/L) and gamma-glutamyl transferase (GGT, 280 U/L).

**Figure 1:**
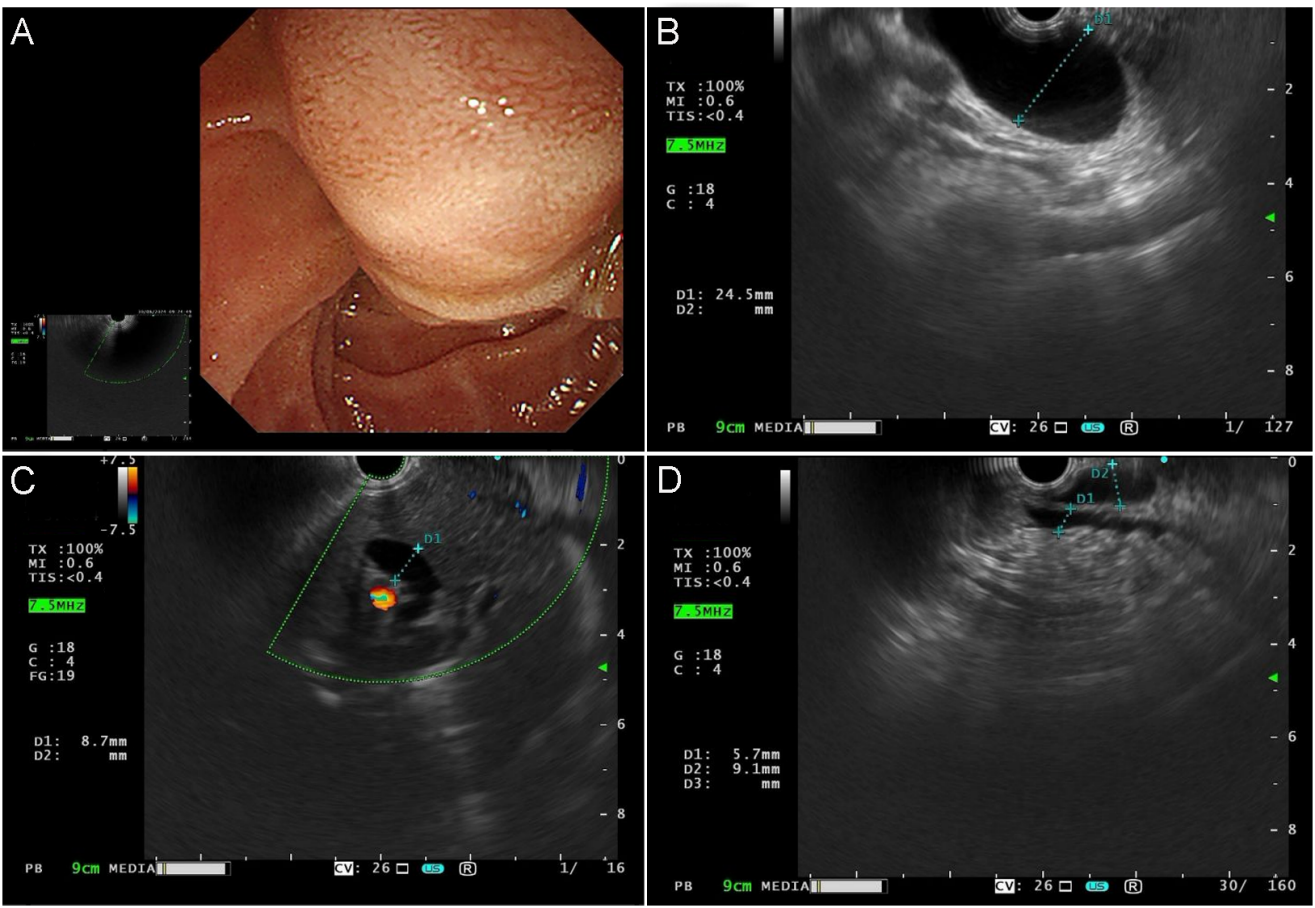
**(A)** Upper gastrointestinal endoscopy shows an enlarged, nodular protrusion at the major duodenal papilla, with a hypoechoic and heterogeneous internal echo pattern. The maximal cross-sectional diameter measures approximately 2.2 × 1.5 cm. Elastography reveals a hard lesion with scant internal vascularity but abundant perilesional blood flow. **(B)** The common bile duct exhibits diffuse dilation, with no abnormal hyperechoic nodules detected within the lumen. The maximal diameter of the common bile duct measures approximately 2.5 cm. **(C)**Marked dilation of the intrahepatic bile ducts is noted, with an internal diameter of approximately 0.7 cm. **(D)** The pancreatic parenchyma demonstrates homogeneous echotexture. The main pancreatic duct is dilated in the pancreatic head segment, with a maximal diameter of approximately 0.7 cm.

**Figure 2:**
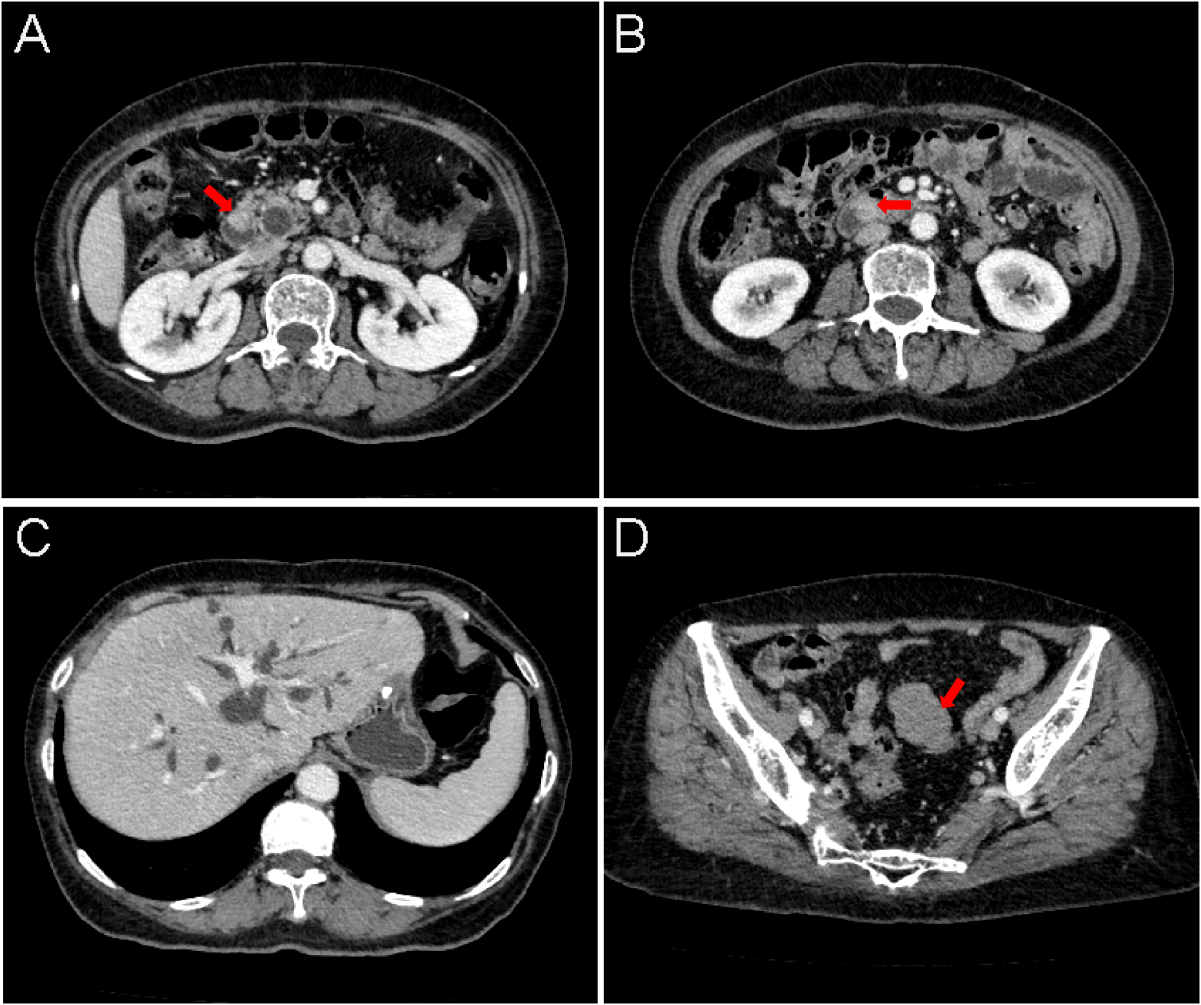
**(A, B)** Soft-tissue density nodules are identified at the orifices of the main and accessory pancreatic ducts in the descending **(A)** and horizontal **(B)** segments of the duodenum, measuring approximately 2.1 cm and 1.9 cm in diameter, respectively. The lesions demonstrate indistinct margins with the pancreatic head and exhibit enhancement characteristics similar to the pancreatic parenchyma on contrast-enhanced imaging (arrow). **(C)** Marked dilation is noted in the main and accessory pancreatic ducts, common bile duct, common hepatic duct, left and right hepatic ducts, and intrahepatic bile ducts. **(D)** A soft-tissue density mass approximately 5 × 3 cm in diameter is visualized superior to the urinary bladder in the pelvic cavity, with poorly defined borders adjacent to the neighboring small intestine (arrow).

**Figure 3:**
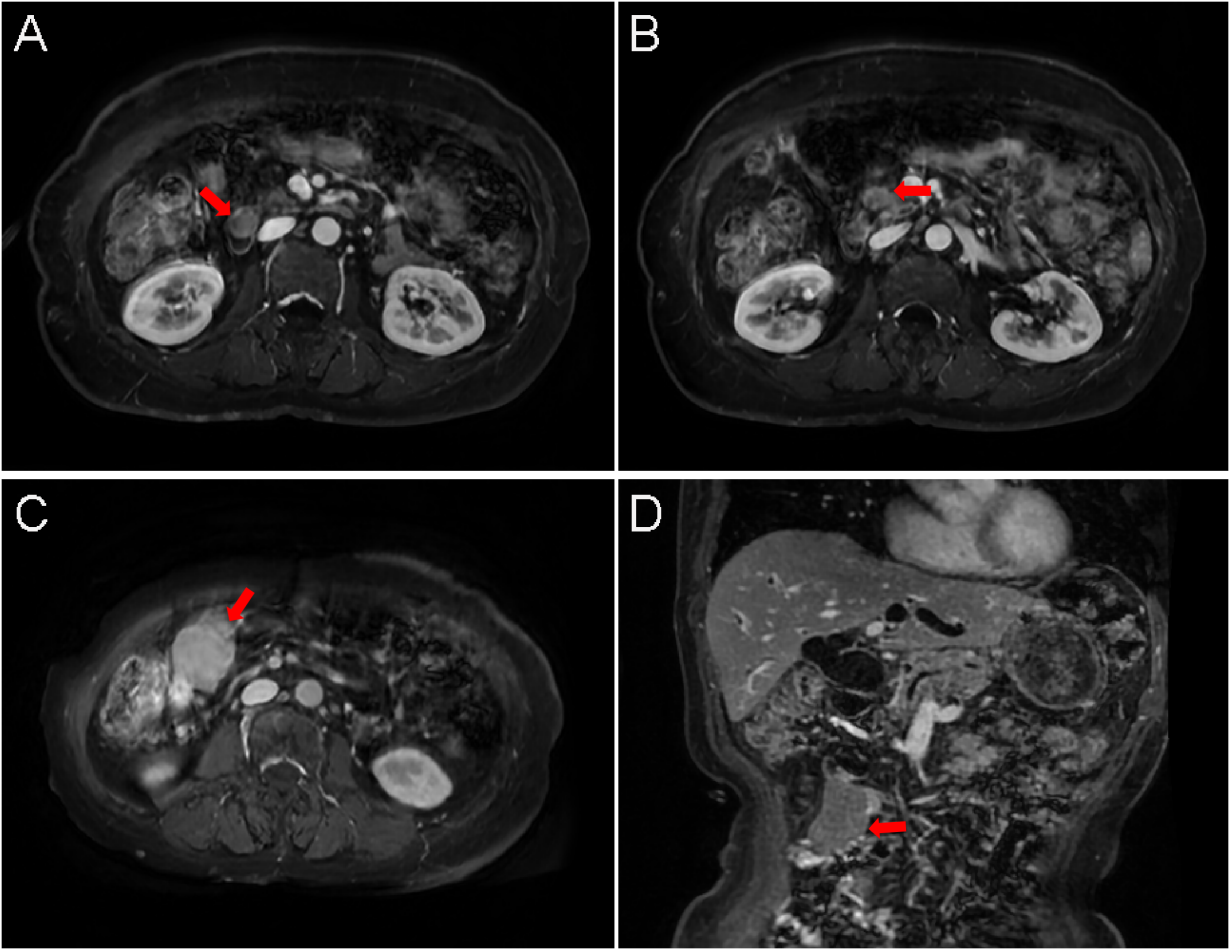
**(A)** The duodenal papilla shows mild enlargement with nodular morphology (maximal longitudinal diameter 1.8 cm), demonstrating subtle restricted diffusion (arrow). Post-contrast imaging reveals mild-to-moderate enhancement. **(B)** A nodular lesion with isointense signal on both T1WI and T2WI is identified within the duodenal bulb lumen, exhibiting lobulated margins (arrow). The lesion shows marked enhancement after contrast administration. **(C, D)** A mass lesion (4.9 × 3.2 cm) with long T1 and long T2 signal characteristics is visualized in the right upper and mid-abdomen (arrow). The mass demonstrates marked enhancement post-contrast and restricted diffusion. Its margins show indistinct demarcation from adjacent small bowel loops.

**Figure 4:**
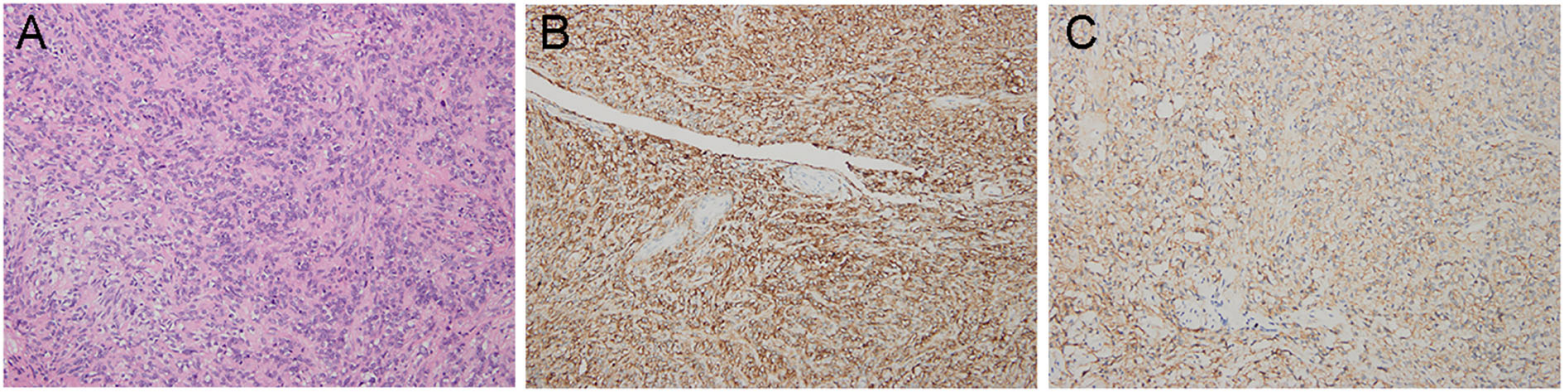
Histopathological examination of the small intestinal neoplasm confirmed the diagnosis of a GIST. **(A)** Hematoxylin–eosin (H&E) staining, magnification ×200. **(B)** Intense immunoreactivity for CD117 (×200.). **(C)** Strong immunohistochemical positivity for DOG1 protein (×200).

Upper gastrointestinal endoscopy identified a mass involving the duodenal papilla (**Figure 1A**). Endoscopic ultrasonography (EUS) revealed a duodenal ampullary neoplasm exerting extrinsic compression and resultant dilatation of both the common bile duct and the main pancreatic duct (**Figures 1B–D**). Fine-needle aspiration biopsy of the mass exhibited diffuse immunoreactivity for synaptophysin (syn) and chromogranin A (cgA), thereby supporting a diagnosis of NET. Contrast-enhanced abdominal CT demonstrated a soft tissue nodule at the confluence of the main and accessory pancreatic ducts within the descending and horizontal duodenum, resulting in ampullary obstruction and significant intra- and extrahepatic biliary dilation (**Figures 2A–C**). A 5 × 3 cm soft-tissue mass was also observed in the pelvic cavity, exhibiting ill-defined margins with adjacent small bowel, suspected to be a small intestinal GIST (**Figure 2D**). Abdominal MRI confirmed nodular enlargement of the duodenal papilla with associated biliary dilation, a markedly enhancing nodule in the duodenal bulb (**Figures 3A, B**), and a persistently enhancing mass in the right upper/mid abdomen with poor demarcation from neighboring small intestine, consistent with a small bowel-originating neoplasm (**Figures 3C, D**). On September 18, 2024, following comprehensive preoperative evaluation. the patient underwent pancreaticoduodenectomy with concurrent partial small bowel resection. Postoperative histopathological analysis demonstrated that the duodenal ampullary neoplasm was a NET of grade 2 (G2), with a ki-67 proliferation index of 3%. The small intestinal neoplasm was composed of spindled and epithelioid cellular elements exhibiting intense immunohistochemical positivity for CD117 and DOG1 (**Figures 4A–C**). The tumor measured 5.7 cm in maximal diameter, with a mitotic count of 9 per 5 mm², findings consistent with a high−risk GIST. Postoperative next-generation sequencing (NGS) identified a germline *NF1* mutation (p.Y2182*). Due to the presence of more than two neurofibromas in the patient, coupled with a pathogenic *NF1* gene mutation identified in peripheral blood cells (allelic variant fraction of 55.63%), the diagnosis of NF1 was performed according the revised diagnostic criteria for NF1 (10). Given complete resection and absence of actionable mutations, adjuvant therapy of imatinib was not administered. As of January 2026, there have been no indications of tumor progression in the patient.

## Discussion

NF1 is a multisystem disorder predisposing individuals to diverse benign and malignant tumors (11). While GISTs, MPNSTs, and neurofibromas are well-documented in NF1, the co-occurrence of a duodenal ampullary NET, high-risk GIST, and gastric leiomyoma is exceptionally rare and previously unreported. This unique case expands the recognized spectrum of NF1-associated neoplasms and emphasizes the critical need for clinical vigilance regarding synchronous or metachronous tumor development in affected individuals.

NF1-associated GISTs demonstrate distinct clinicopathological features compared to their sporadic counterparts. These tumors typically arise in the small intestine and notably lack the characteristic *KIT* or *PDGFRA* mutations seen in sporadic cases. Instead, tumorigenesis appears driven through dysregulation of the RAS/MAPK pathway secondary to *NF1* gene inactivation (12). These tumors are often multiple, small, and located in the jejunum or ileum, and although they tend to follow an indolent course, they can exhibit progressive growth and lead to complications such as bleeding or obstruction (13, 14). In the present case, despite the NF1-associated origin, the tumor was classified as ​high-risk GIST, with a ​mitotic count of 9 per 5 mm², indicating a ​more aggressive biological behavior.

Concurrently, the patient was found to have a NET located in the duodenal ampulla, a relatively uncommon site for such neoplasms. Duodenal NETs, although rare compared to those originating in the pancreas or rectum, are clinically significant due to their potential to cause biliary or pancreatic duct obstruction, leading to symptoms such as jaundice or pancreatitis. These tumors are classified by the WHO system based on tumor grade, which incorporates mitotic count and Ki-67 index (15). In the present case, histological examination confirmed a grade 2 (G2) NET with moderate proliferative activity. The therapeutic approach comprised pancreaticoduodenectomy, which remains the standard intervention for localized, resectable ampullary NETs.

In addition to the GIST and NET, the patient had a documented history of gastric leiomyoma, a benign smooth muscle tumor, which had been resected six years prior along with a cutaneous neurofibroma. Although the association between gastric leiomyomas and NF1 is not as clearly defined as that with GISTs or NETs, the presence of this lesion further illustrates the patient’s long-standing predisposition to a variety of neoplastic processes. The coexistence of three distinct tumor types, two of mesenchymal origin (GIST and leiomyoma) and one neuroendocrine, highlights the complex tumor biology in NF1 and suggests a possible broader susceptibility to tumorigenesis across different cellular lineages.

NGS performed on the resected specimens identified a germline *NF1* mutation: p.Y2182*. This pathogenic variant likely serves as the underlying genetic driver for the patient’s tumor predisposition and is consistent with the known role of *NF1* as a tumor suppressor gene involved in regulating the RAS signaling pathway (16). Germline mutations in *NF1* are associated with a highly variable clinical phenotype, and the presence of multiple primary tumors in a single individual, as seen in this case, reflects the profound impact of *NF1* dysfunction on cellular growth regulation. Importantly, the identification of a germline mutation also has implications for genetic counseling and the evaluation of at-risk family members.

Current literature regarding NF1-associated tumors primarily focuses on isolated neoplasms, with limited documentation of complex multi-tumor presentations. This case represents the first report of synchronous duodenal ampullary NET, high-risk GIST, and previous gastric leiomyoma in an NF1 patient, thereby expanding the recognized phenotypic spectrum of this disorder. The management of such complex cases requires a multidisciplinary approach involving gastroenterologists, surgeons, oncologists, pathologists, and geneticists. In our patient, complete surgical resection of both the duodenal ampullary NET and the small intestinal GIST was achieved through pancreaticoduodenectomy and partial small bowel resection, respectively. Given the absence of residual disease and the lack of actionable molecular targets (such as *KIT* or *PDGFRA* mutations in the GIST), no adjuvant therapy was administered. The patient remains under close follow-up with no evidence of recurrence to date. This outcome underscores the importance of early detection, complete surgical extirpation, and long-term surveillance in optimizing outcomes for patients with NF1 and associated neoplasms.

## Conclusion

In conclusion, this report describes a unique case of synchronous duodenal ampullary NET, high-risk GIST, and gastric leiomyoma in an NF1 patient, highlighting the complex tumor spectrum associated with this genetic disorder. Comprehensive diagnostic workup, genetic testing, and multidisciplinary management are essential for addressing such rare and multifaceted presentations. Long-term surveillance is recommended to detect potential recurrence or new primary tumors timely.

## Data Availability

The original contributions presented in the study are included in the article/supplementary material. Further inquiries can be directed to the corresponding authors.
